# Supplementary material for: Eye tracking and eye expression decoding based on transparent, flexible and ultra-persistent electrostatic interface
Source: Nat Commun. 2023 Jun 7;14:3315. doi: 10.1038/s41467-023-39068-2 (PMC10247702; doi:10.1038/s41467-023-39068-2)
Supplement: Supplementary file 1 — Supplementary Information [file 41467_2023_39068_MOESM1_ESM.pdf]

Supplementary Materials for

Eye tracking and eye expression decoding based on transparent, flexible and ultra-persistent electrostatic interface

Yuxiang Shi[1,2], Peng Yang[1,2], Rui Lei[1], Zhaoqi liu[1,2], Xuanyi Dong[1,2], Xinglin Tao[1,2], Xiangcheng Chu[3], Zhong Lin Wang[1,3], Xiangyu Chen*[1,2]

[1]CAS Center for Excellence in Nanoscience, Beijing Key Laboratory of Micro-nano Energy and Sensor, Beijing Institute of Nanoenergy and Nanosystems, Chinese Academy of Sciences, Beijing 100083, China.
[2]School of Nanoscience and Technology, University of Chinese Academy of Sciences, 100049 Beijing, China.
[3]State Key Laboratory of New Ceramics and Fine Processing, Tsinghua University, Beijing 100084, China
[4]Georgia Institute of Technology, Atlanta, GA 30332-0245, USA.
*Corresponding author: X.C. (chenxiangyu@binn.cas.cn).

This file includes: Supplementary Fig. 1 to 24, Supplementary Table 1 to 4, Supplementary Note 1 to 3.

Other Supplementary Materials for this manuscript include the following:
Supplementary Movie 1. Eye movement patterns.
Supplementary Movie 2. Closing eye tracking.
Supplementary Movie 3. Eye tracking system for visual preference analysis.
Supplementary Movie 4. Eye-controlled input modality.

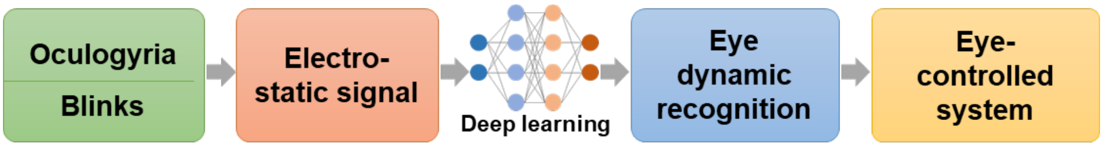

Supplementary Fig. 1. Overview process of the eye-controlled input modality for human-computer interaction.

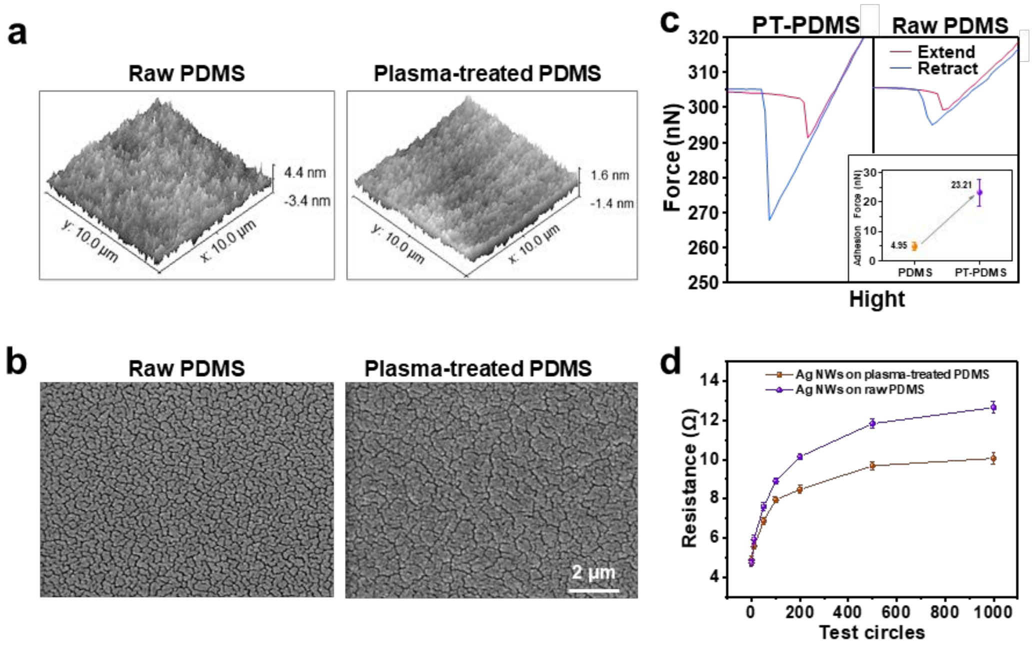

Supplementary Fig. 2. Plasma-treated PDMS. (**a**–**c**) AFM images for dimensional surface morphology (**a**), SEM images (**b**) and force curves with insert adhesion force (**c**) on raw PDMS with and plasma-treated PDMS (PT-PDMS). The insert data are presented as mean (SD) with error of repeated tests: n = 5. (**d**) Surface resistance of bended Ag NW electrode on raw and plasma-treated PDMS. Data are presented as mean (SD) with error of repeated tests: n = 5.

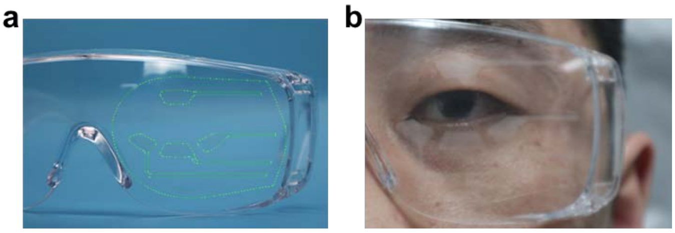

Supplementary Fig. 3. (a and b) Digital photos of the interface array attached on glasses (a) and worn by participant (b).

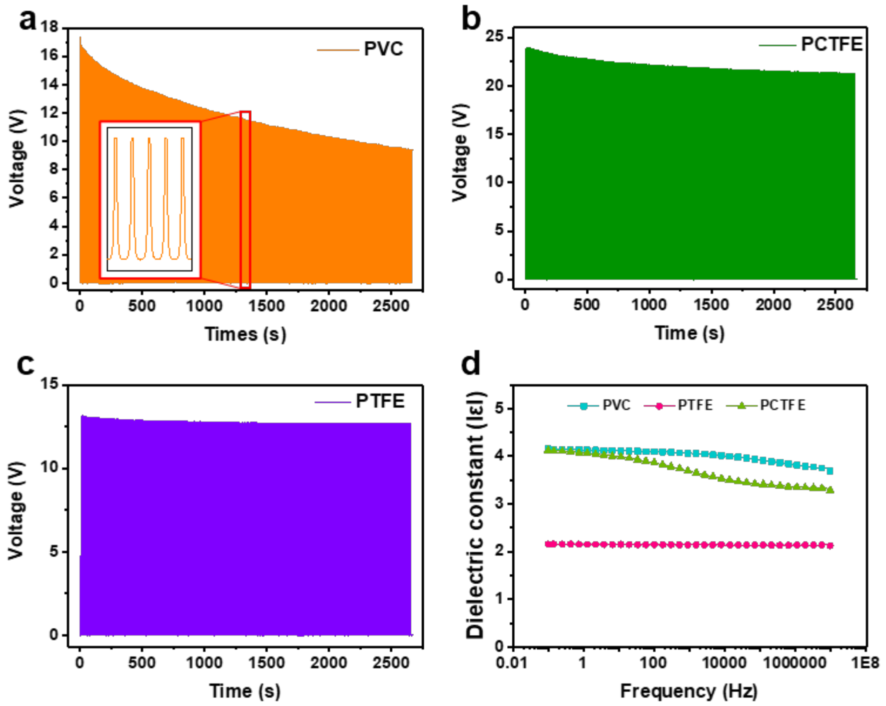

Supplementary Fig. 4. Output performance and dielectric property of PVC, PTFE and PCTFE. (**a**–**c**) The open-circuit voltage of PVC (**a**), PCTFE (**b**) and PTFE (**c**) under non-contact condition. Repeat times: 1000. (**d**) Dielectric permittivity spectra of the three materials.

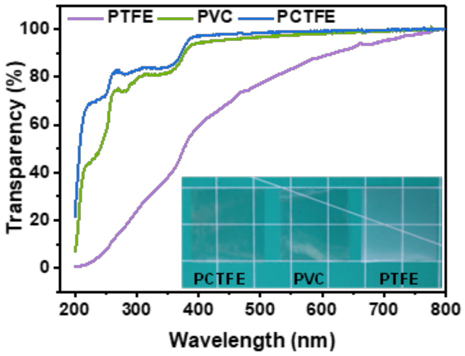

Supplementary Fig. 5. UV-Vis transmittance spectra of PTFE, PVC and PCTFE films (Thickness: 30 μm). Insert: Digital photo of the three films.

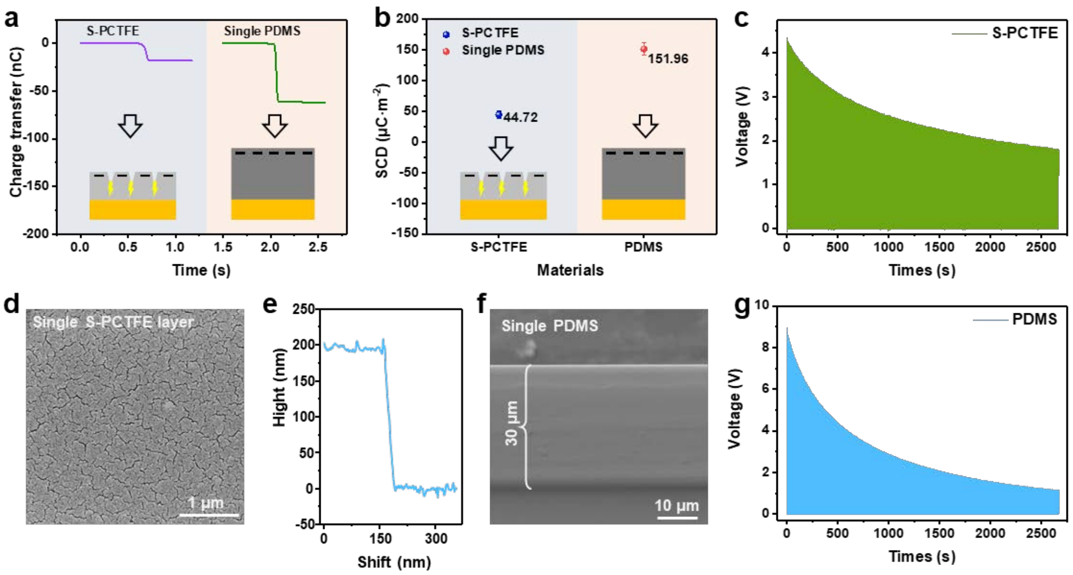

Supplementary Fig. 6. Output performance and characterization of single layer of sputtered PCTFE (S-PCTFE) and PDMS. (**a** and **b**) Transferred charge (**a**) and average surface charge density (**b**) of S-PCTFE and PDMS. (**c**) Open circuit voltage of S-PCTFE under non-contact condition. (**d**) SEM image of S-PCTFE. (**e**) Height curve of S-PCTFE tested by a step meter. (**f**) Cross section view of the single PDMS layer. (**g**) Open circuit voltage of PDMS under non-contact condition. Repeat times: 1000.

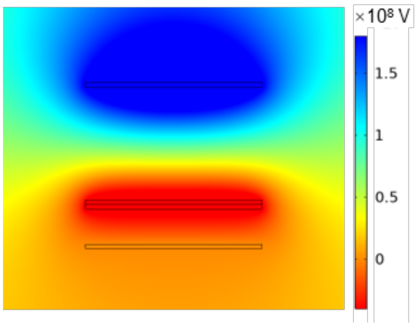

Supplementary Fig. 7. A simulated model established for the interface. A positively charged (0.001 C·m^2) film away from the negatively charged PTFE (-0.001 C·m^2) with a back Cu electrode and another Cu electrode as grounded electrode.

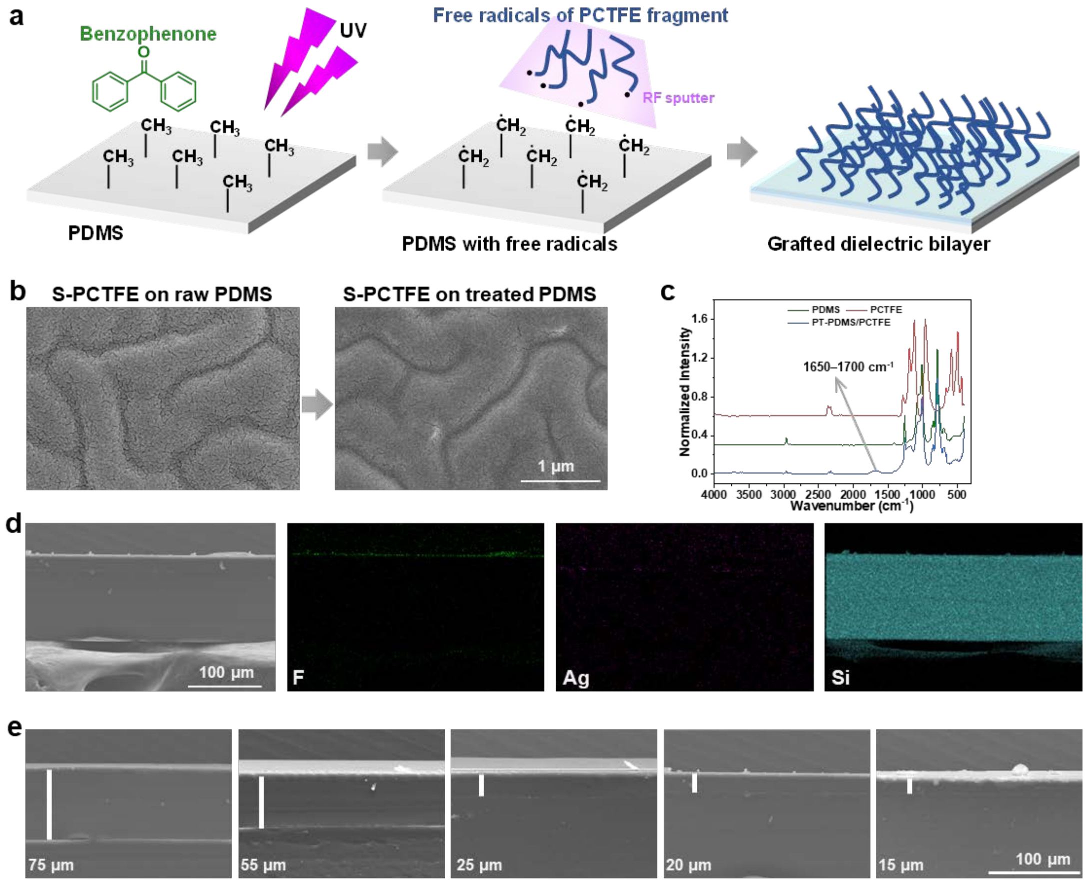

Supplementary Fig. 8. Fabrication and characterization of the dielectric bilayer. (**a**) Fabrication progress of the bilayer. (**b**) Nano morphology of S-PCTFE sputtered on raw and benzophenone-treated PDMS. (**c**) ATR-FTIR spectra of PDMS, PCTFE and the bilayer (PT-PDMS/PCTFE). (**d**) Energy Dispersive Spectroscopy image (Cross section) of the layered interface (Elements: **F**, **Ag**, **Si**). (**e**) Cross section of the dielectric bilayer layer with various thickness controlled by spin coating speed.

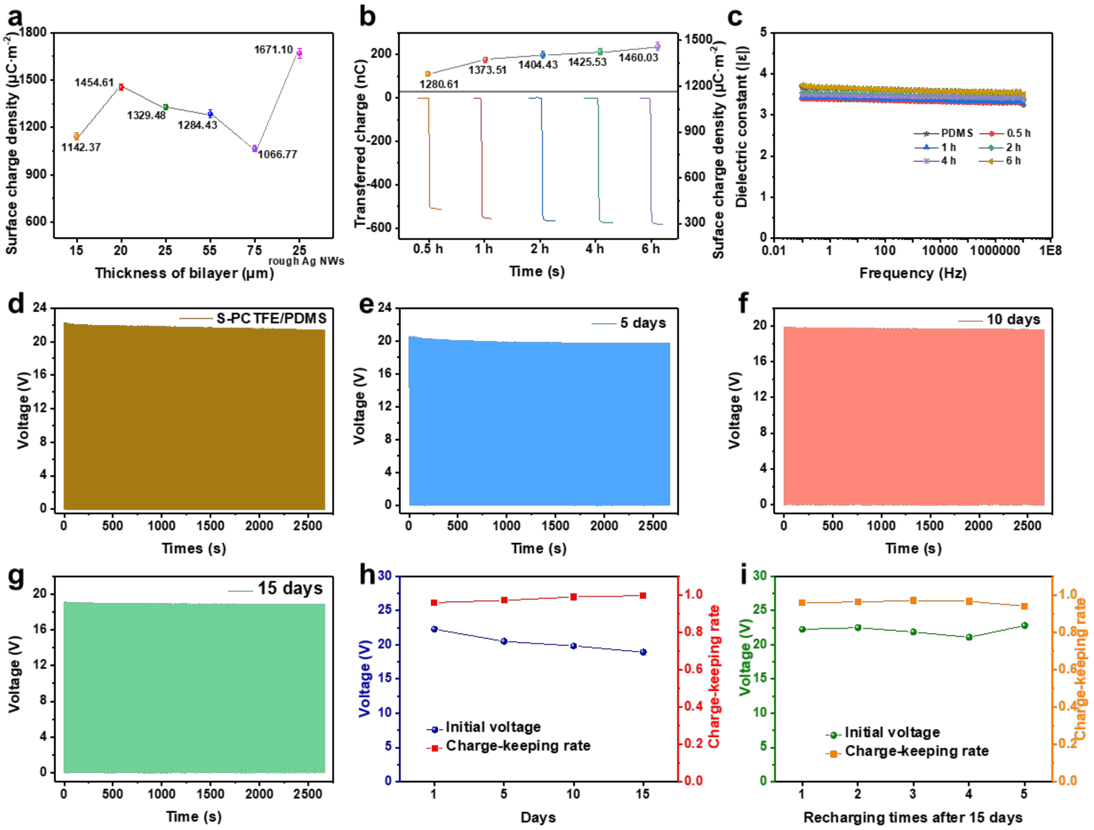

Supplementary Fig. 9. Output performance of the bilayer. (**a**) Surface charge density of the dielectric bilayer with various thickness (15, 20, 25, 55, 75, and 20 with rough electrode). Data are presented as mean (SD) with error of repeated tests: n = 5. (**b** and **c**) Output performance (**b**) and dielectric property (**c**) of the dielectric bilayer with various sputtering time of PCTFE (depositing thickness 50 nm/h) on PDMS (thickness: 20 μm). Data are presented as mean (SD) with error of repeated tests: n = 5 (**d**) The open-circuit voltage of the bilayer (S-PCTFE/PDMS). (**e**–**g**) Open-circuit voltage of the bilayer after 5 days (**e**), 10 days (**f**) and 15 days (**g**). (**h**–**i**) Initial voltage and charge keeping rate of the bilayer after various days (**h**) and recharged after 15 days (**i**).

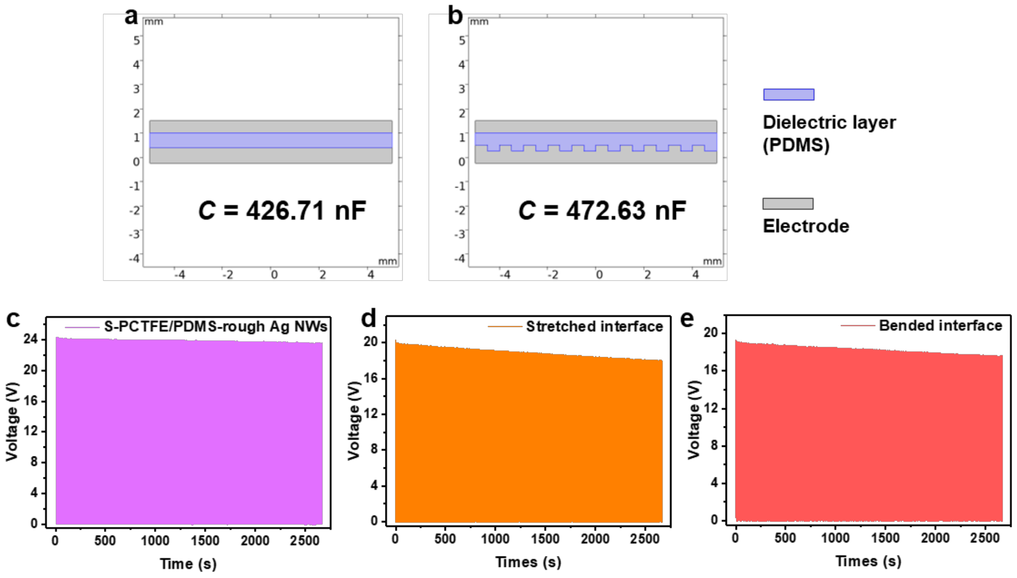

Supplementary Fig. 10. Rough Ag NWs modified interface. (**a** and **b**) The simulated model to calculate the capacitance of dielectric bilayer on smooth (**a**) and rough (**b**) electrode with the same average thickness. (**c**–**e**) Open circuit voltage of the raw (**c**), stretched (**d**) and bended (**e**) interface under non-contact condition. Repeat times: 1000.

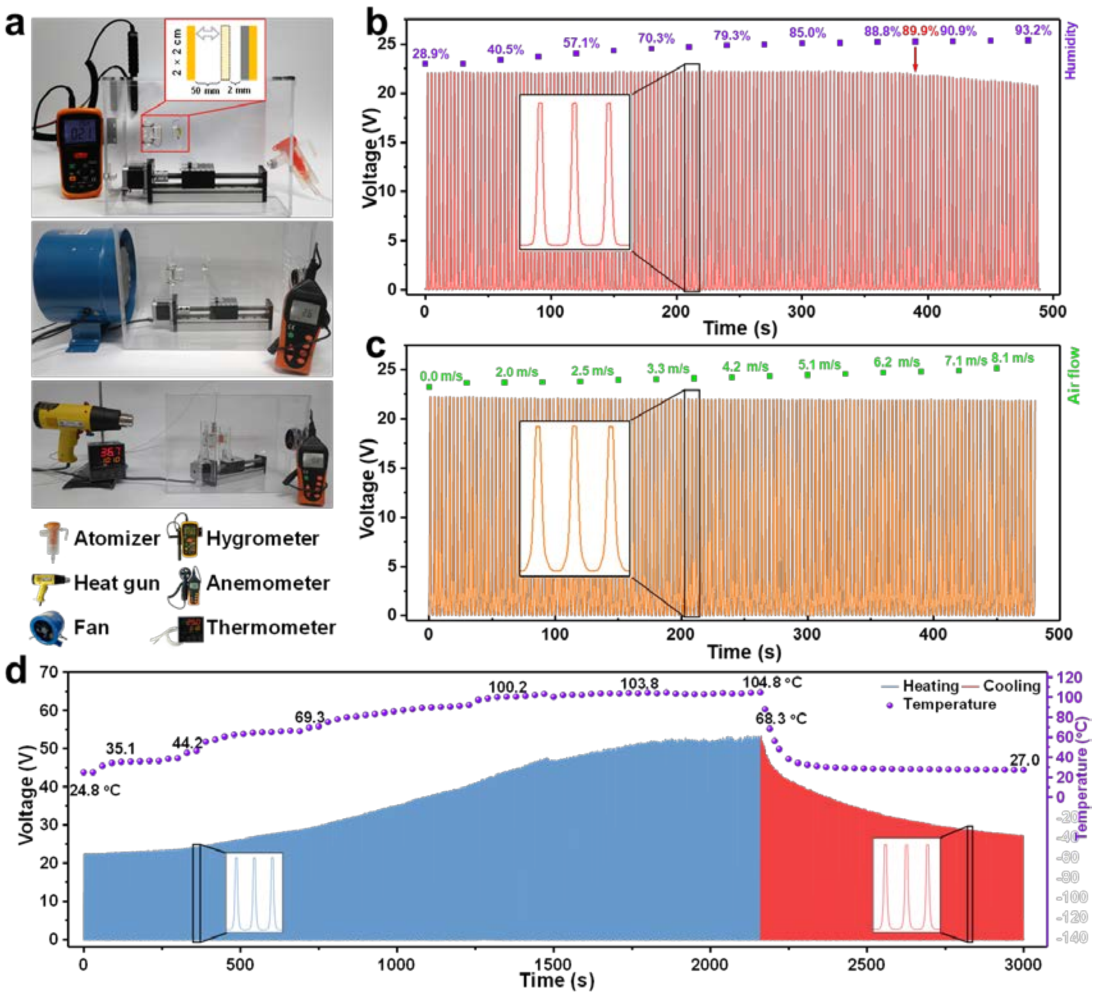

Supplementary Fig. 11. Stability test of the interface under environmental factors. (**a**) Experimental devices for testing the stability of the interface under various humidity (I), air flow (II) and temperature (III). (**b** and **c**) Open-circuit voltage of the interface under increased humidity (**b**) and air flow (**c**). (**d**) Open-circuit voltage of the interface under heating and cooling conditions.

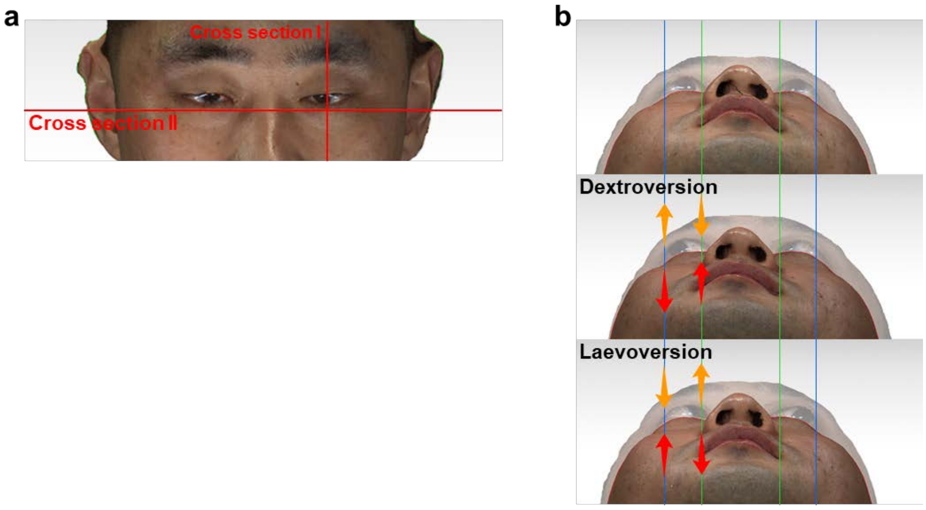

Supplementary Fig. 12. 3D face scan image3D face scan images. (**a**) The cross section I and II marked for analyzing vertical and horizontal eye movements. (**b**) 3D face scan image of dextroversion and laevoversion of corss section II.

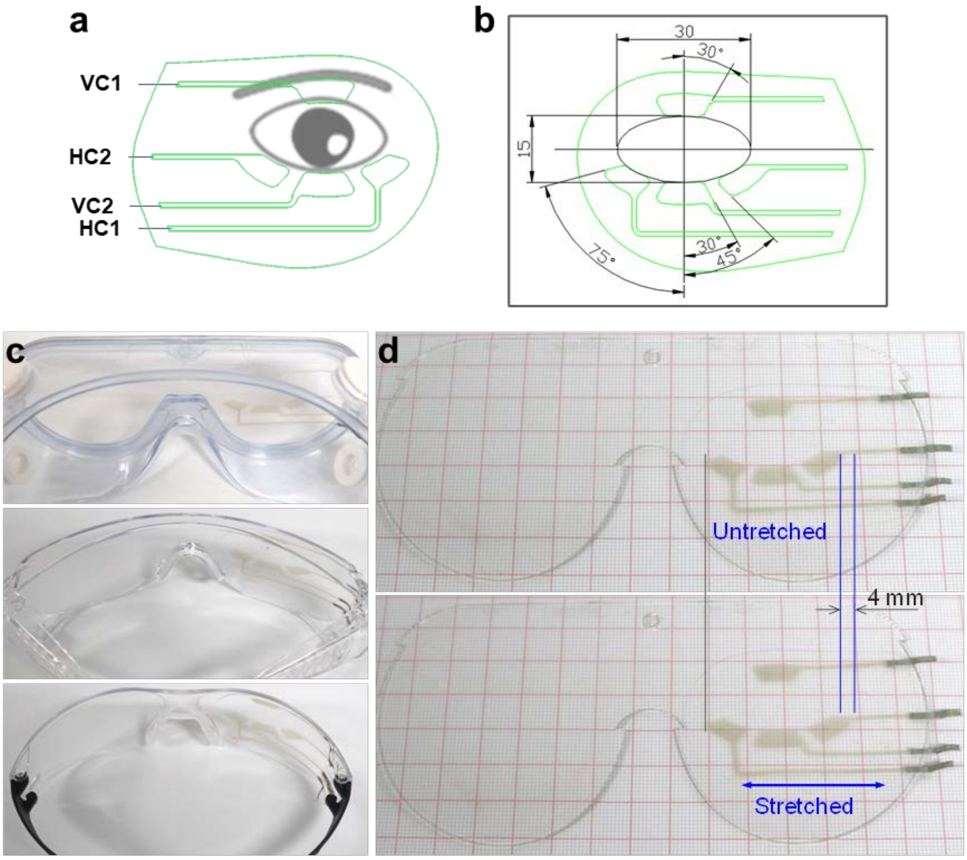

Supplementary Fig. 13. The electrostatic interface array. (**a** and **b**) The configuration (**a**) and annotation figure (**b**) of the interface array with four channels: vertical channel 1 (VC1), vertical channel 2 (VC2), horizontal channel 1 (HC1) and horizontal channel 2 (HC2). (**c**) The flexible interface adhered on glasses with various curvature. (**d**) The stretched interface adhered on a pair of glasses.

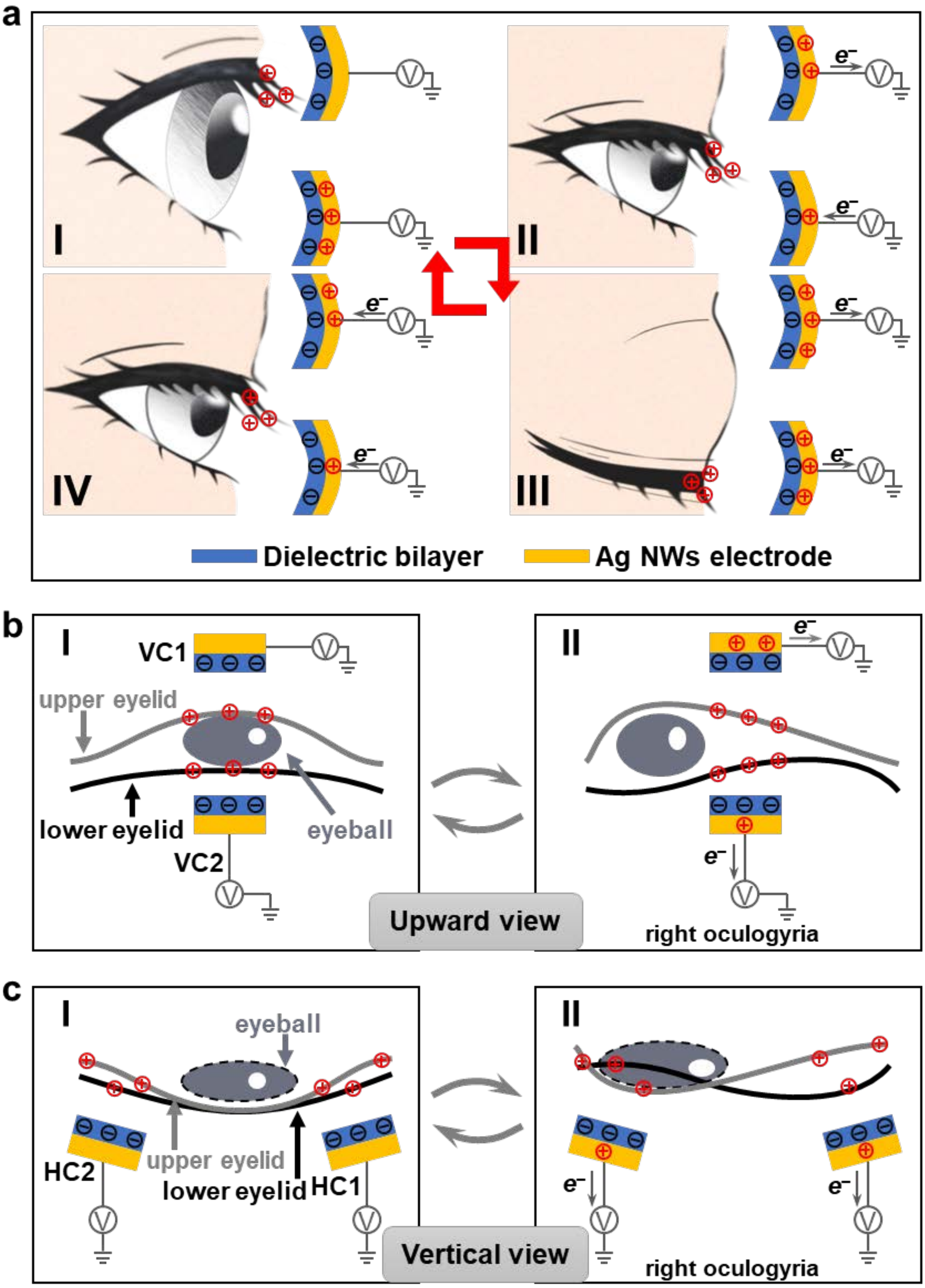

Supplementary Fig. 14. Mechanism analysis of the interface array. (**a**) Mechanism responding to a blink. (**b**–**c**) Mechanism responding to right oculogyria with upward view (**b**) and vertical view (**c**).

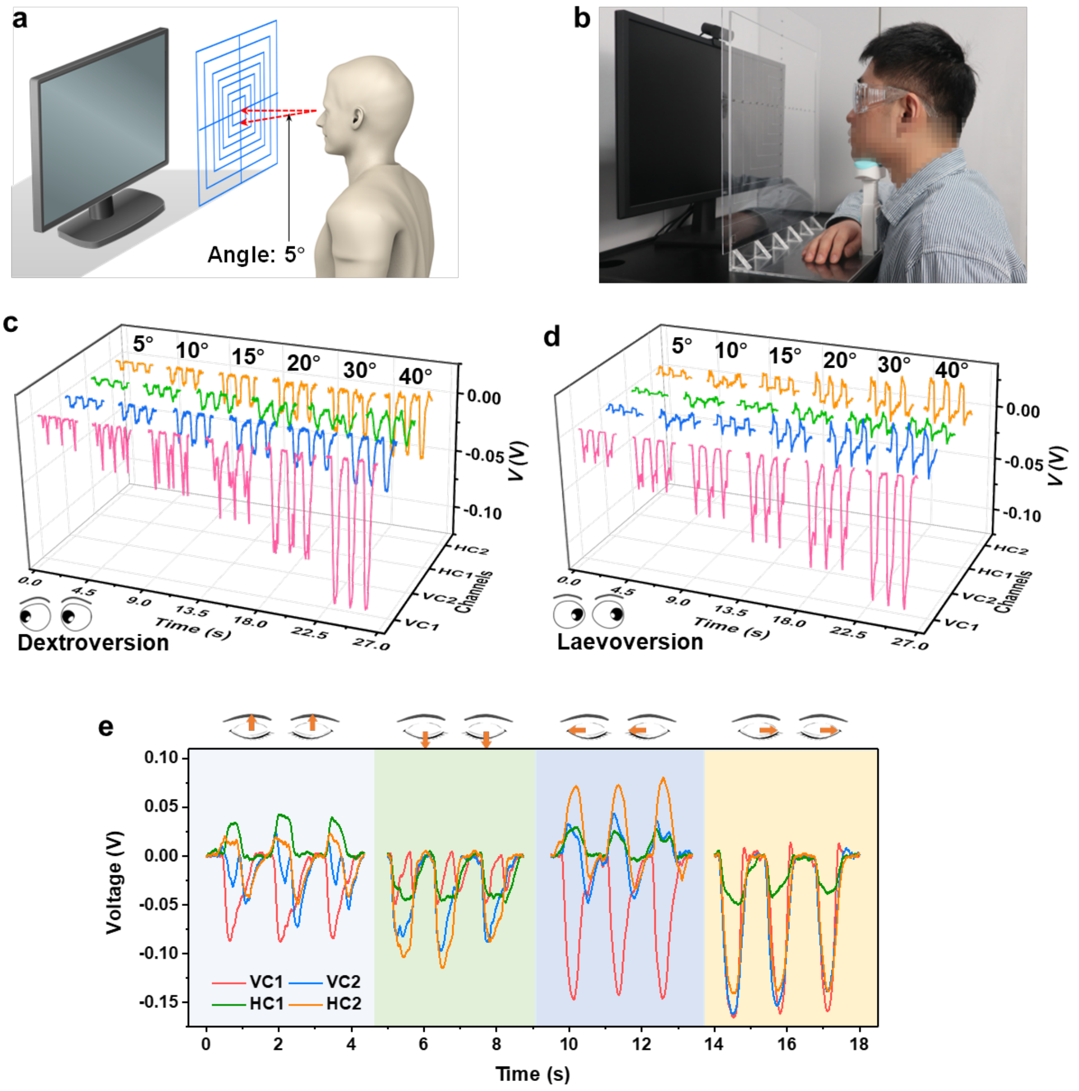

Supplementary Fig. 15. Eye tracking of eyeball rotation at various degrees and closing eyes. (**a** and **b**) Controlled degree of eyeball rotations with a posture controlling the head and a transparent scale board for controlling the degree: diagrammatic sketch (**a**) and testing photograph (**b**). (**c** and **d**) Electrostatic signals of dextroversion (**c**) and laevoversion (**d**) at degrees from 5° to 40°. (**e**) Electrostatic signals of closing eye moving at various directions.

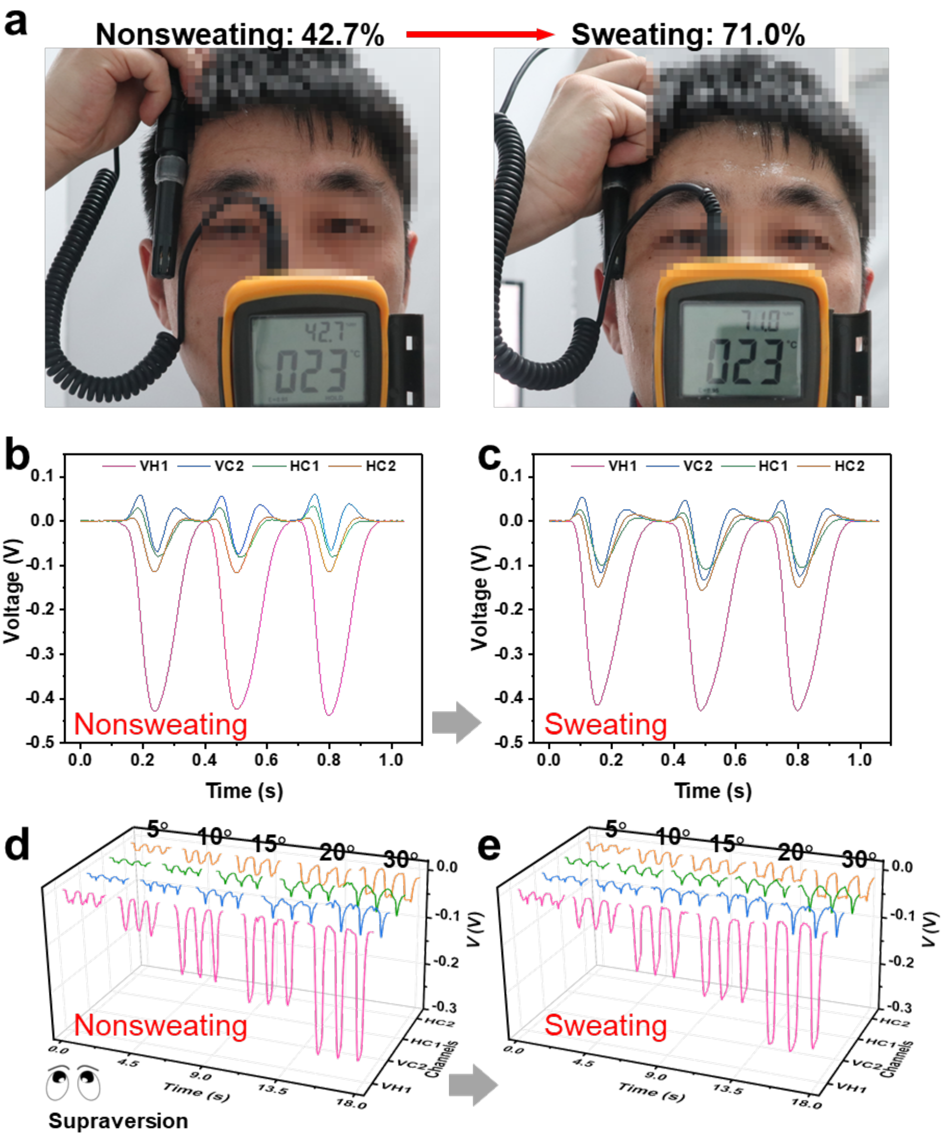

Supplementary Fig. 16. Eye-tracking performance under sweating. (**a**) Periocular humidity test after sweating. (**b** and **c**) Blink signals under nonsweating (**b**) and (**c**) sweating conditions. (**d** and **e**) Supraversion signals under nonsweating (**d**) and (**e**) sweating conditions.

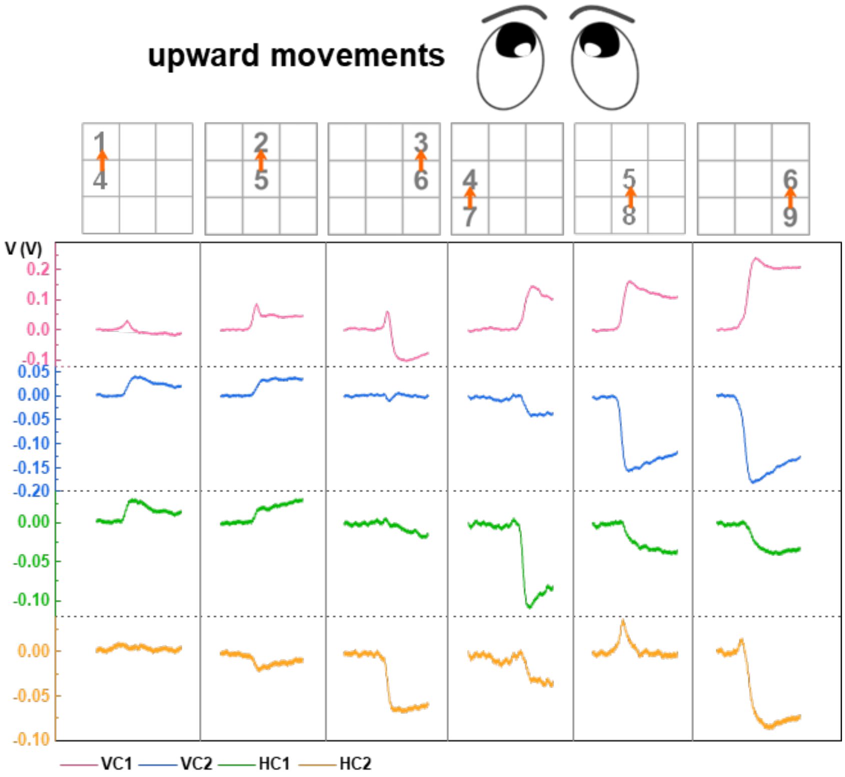

Supplementary Fig. 17. Decoding upward eye movements in the paths (4→1, 5→1, 6→3, 7→4, 8→5, 9→6) and related electrostatic signals.

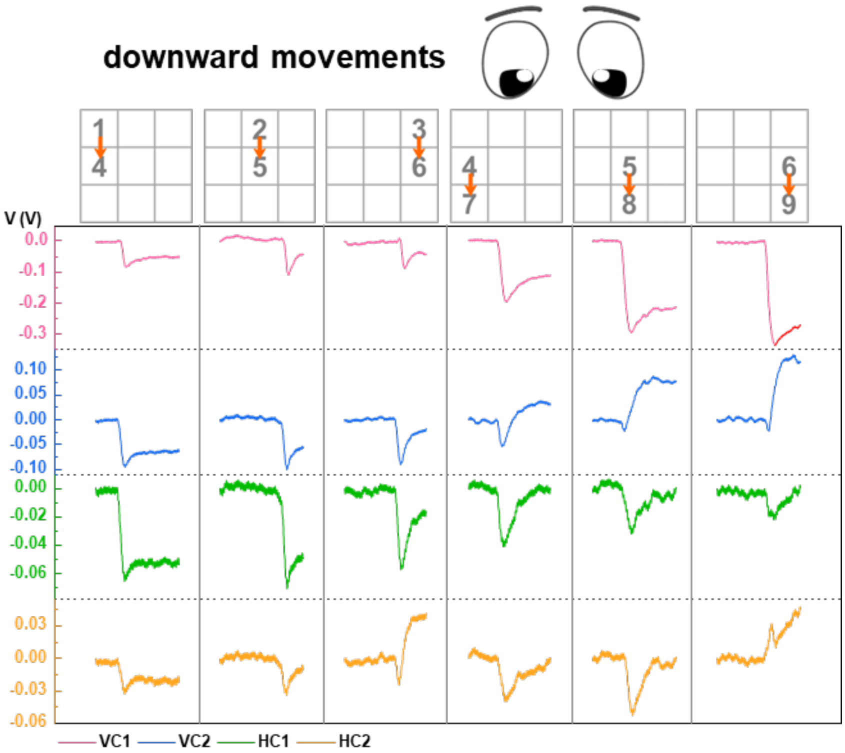

Supplementary Fig. 18. Decoding downward eye movements in the paths (1→4, 2→5, 3→6, 4→7, 5→8, 6→9)4 and related electrostatic signals.

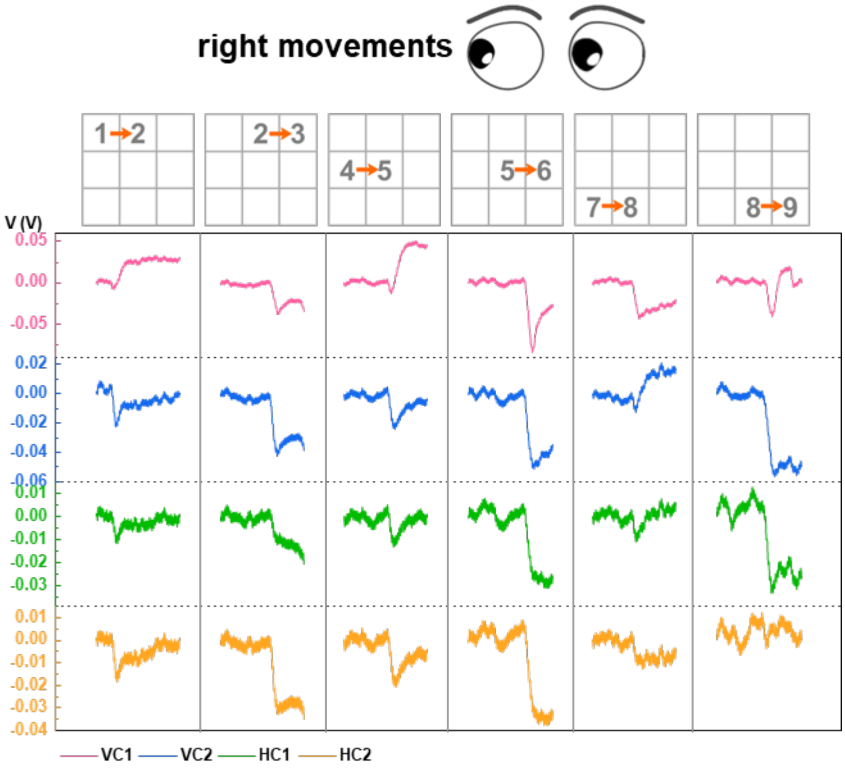

Supplementary Fig. 19. Decoding right eye movements in the paths (1→2, 2→3, 4→5, 5→6, 7→8, 8→9) and related electrostatic signals.

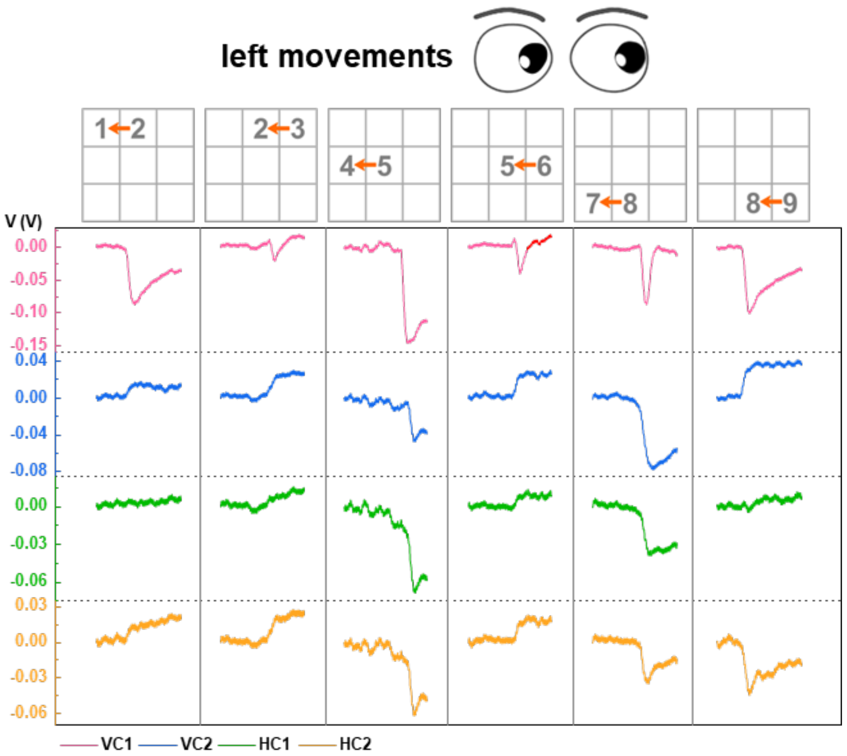

Supplementary Fig. 20. Decoding left eye movements in the paths (2→1, 3→2, 5→4, 6→5, 8→7, 9→8) and related electrostatic signals.

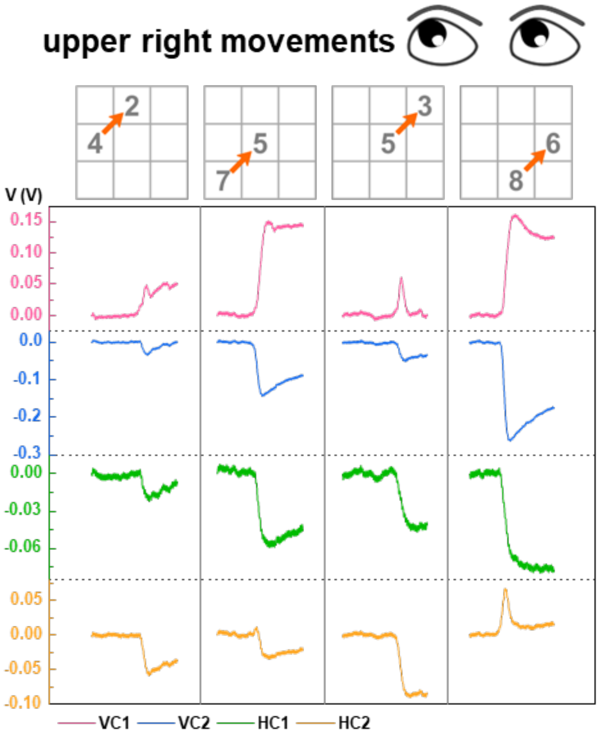

Supplementary Fig. 21. Decoding upper right eye movements in the paths (4→2, 7→5, 5→3,8→6) and related electrostatic signals.

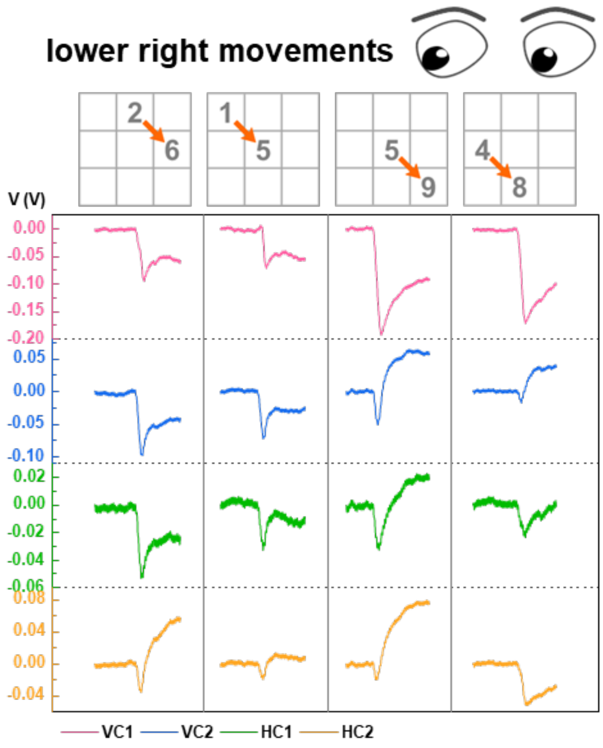

Supplementary Fig. 22. Decoding lower right eye movements in the paths (2→6, 1→5, 5→9, 4→8) and related electrostatic signals.

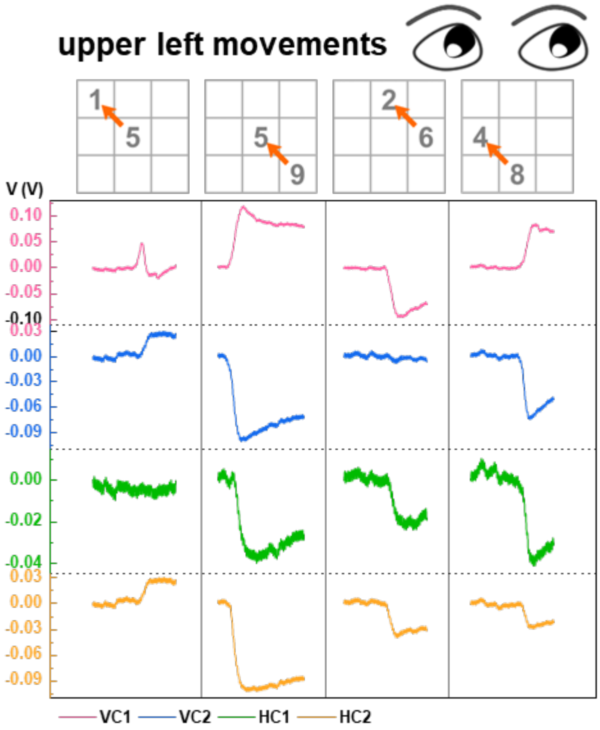

Supplementary Fig. 23. Decoding upper left eye movements in the paths (5→1, 9→5, 6→2, 8→4) and related electrostatic signals.

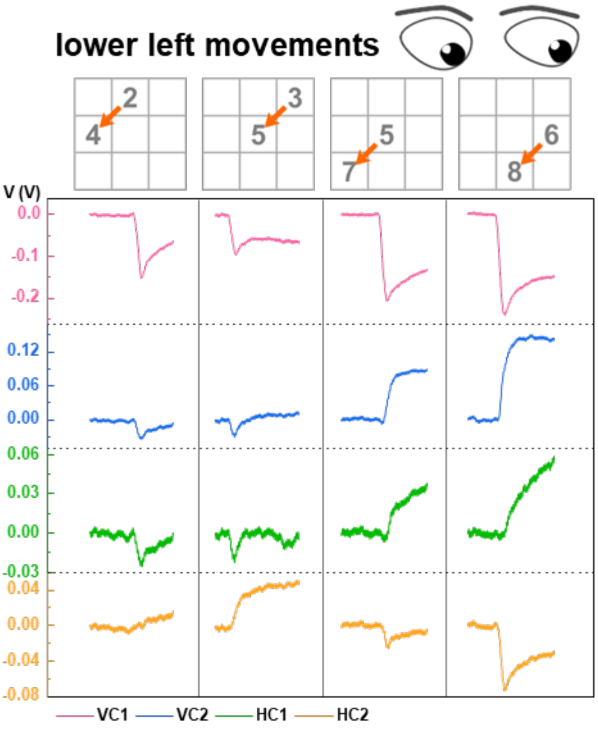

Supplementary Fig. 24. Decoding upper right eye movements in the paths (2→4, 3→5, 5→7, 6→8) and related electrostatic signals.

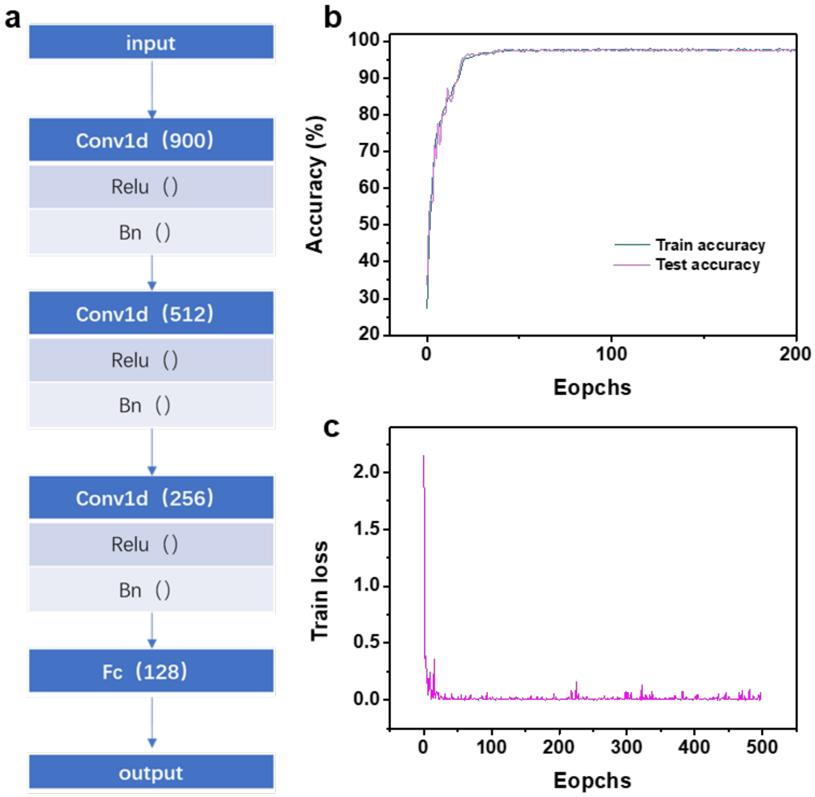

Supplementary Fig. 25. Deep learning of the eye movements and gazing tracking system. (**a**) Neural network module of deep learning utilized in the system. (**b** and **c**) Accuracy (**b**) and train loss (**c**) curves of the network.

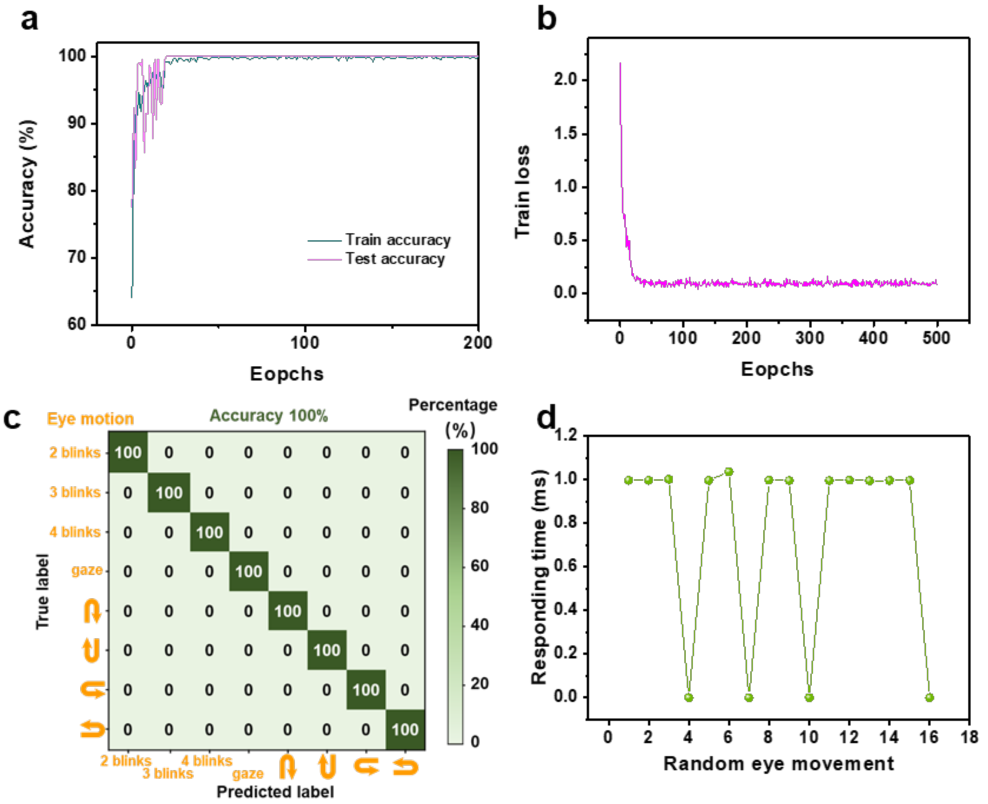

Supplementary Fig. 26. Deep learning of the eye-controlled input modality. (**a** and **b**) Accuracy (**a**) and train loss (**b**) curves of the network. (**c**) Confusion map of deep learning outcomes. (**d**) Responding time from signal input to movement decoding (random action in Fig. 5c–d).

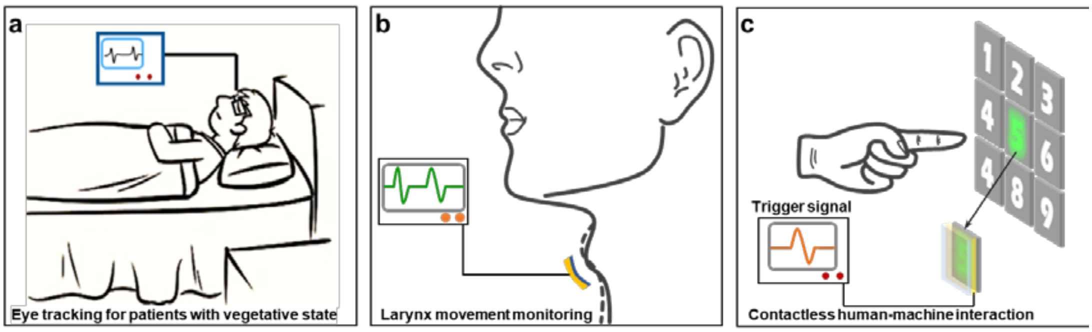

Supplementary Fig. 27. Potential application of the electrostatic interface. (**a**) REM sleep monitoring. (**b**) Larynx movement monitoring. (**c**) Contactless human-machine interactions.

Directions	XX	YY	ZZ	Average	Volume resistivity (ohms·cm)
PTFE	137.43	97.91	99.60	111.65	1E+18
PVC	176.17	152.71	133.79	154.23	13E+13
PCTFE	177.63	152.74	144.43	158.27	2E+16

Supplementary Table 1. Calculated molecular polarizability and volume resistivity (from handbook) of PTFE, PVC and PCTFE.

Directions	XX	YY	ZZ	Average
C–F	7.90	8.34	11.24	9.16
C–Cl	11.79	11.27	29.56	17.54

Supplementary Table 2. Calculated molecular polarizability of C–F and C–Cl.

Humidity (%)	Air flow (m/s)	Temperature (°C)
100	10	100
90	9	90
80	8	80
70	7	70
60	6	60
50	5	50
40	4	40
30	3	30
20	2	20
10	1	10
0	0	0

Stable operation range

Supplementary Table 3. The available range (including factors of humidity, air flow and temperature) of the electrostatic interface.

Eye-tracking method	Mechanism	Main parts	Advantages	Disadvantages	Optimal applications
Video oculography (VOG)	Video-recorded eye movement	Recording cameras	• Uncomplicated settings<br>• Inexpensive	• Limited spatial resolution<br>• Privacy risks	Commercial analysing<br>Virtual reality
Infrared oculography (IOG)	Sclera-mirrored infrared light	Infrared light receiver	• Used in light and darkness	• Unable to torsional eye<br>• Invasive method	Commercial analysing
Magnetic resonance-based tracker	Magnetic resonance	Magnetic resonance imaging scanners	• Brain activities analysing simultaneously	• rely independently on cumbersome equipment	Medical fields
Scleral search coil	Magnetic induction	Invasive coil contact lenses	• High temporal and spatial resolution	• Invasive method<br>• Complicated settings	Medical fields
Electrooculography (EOG)	Corneo-retinal standing potential	5 skin-integrated electrodes	• High temporal and spatial resolution	• Not used daily<br>• Complicated settings	Medical fields and laboratories
TENG-based tracker	Triboelectrification and electrostatic induction	Layered structure of dielectric layer and electrode films	• Simple structure, inexpensive<br>• Closing eye tracking<br>• Noninvasive	• Limited spatial resolution	Wearable electronics<br>Virtual reality

Supplementary Table 4. A summary of eye tracking methods with their advantages and disadvantages.

References	Mechanism	Blinks	Resolution		Diagonal movement	Accuracy	Contact (Y/N)	Reported applications
			Vertical	Horizontal				
[1]	EOG	√	√	√	×		Y	Grid game
[2]	EOG	√	×	×	×	96%	Y	
[3]	EOG	×	10°	10°	×		Y	
[4]	EOG	√	×	10°	×		Y	Tetris game
[5]	EOG	√	√	√	×	80.32%	Y	Whack a mole game
[6]	EOG	√	×	15°	×	92.6%	Y	
[7]	EOG	×	√	√	√		Y	
[8]	EOG	×	√	√	×		Y	Eye-controlled quadcopter
[9]	EOG	√	4°	4°	×		Y	Eye-controlled quadcopter
[10]	EOG	√	√	√	×		Y	Eye-controlled Snake game
[11]	Capacitive	√	√	√	×		N	
[12]	Piezoelectric	×	×	√	×		Y	
[13]	Triboelectric	√	√	√	×		Y	Eye-controlled T9 input
[14]	Triboelectric	√	×	×	×		Y	Driver fatigue monitoring
[15]	Triboelectric	×	√	√	√		Y	
[16]	Triboelectric	√	×	×	×		Y	REP sleep monitoring
This work	Triboelectric	√	5°	5°	√	97%	N	Mouse input;<br>Commercial analysis<br>REP sleep monitoring

Supplementary Table 5. Comparation of electric signal-based eye tracker between previous studies and this work.

Supplementary Note 1:

Mechanism analysis of the interface array to eye dextroversion.

As is shown in an upward view (Supplementary Fig. 13b), the outlines of eyelid are depicted base on the Supplementary Fig. 11b and Movie 1. The periocular skin is supposed positive charged to equilibrate the negative interface. From stage I to II, it is obviously that the center parts of upper and lower eyelid are away from the channel of VC1 and VC2, which cause the electrons flow out of the back electrodes resulting the negative voltage in VC1 and VC2. As for the area opposite to HC2, as shown in Supplementary Fig. 13c (a vertical view), when eyeball turning right, the right side of the lower eyelid is concave to close to HC2, but the right side of the upper is convex, and the final effect on HC2 is a negative potential signal. It is totally contrary of HC1, the convex lower eyelid and concave upper eyelid is observed and finally it results in a negative potential on HC1. Expressly, the actual interactions between periocular skin and the interface are beyond of this simplified illustration, Thus, the signal of dextroversion has some differences with that of laevoversion (Fig. 3c, Supplementary Fig. 14c and d). Besides, the number of electrons in the figure is just qualitatively describe the direction of the potential but not quantitatively.

Supplementary Note 2:

Basic setup of the eye-tracking system.

a. The participant is required to move eyes in the neighboring grid in horizontal, vertical and diagonal directions.
b. The deep learning progress extract data of the electrostatic signal every 2 s, and every eye movement of the participant are required to finished within 2 s, which is reminded by a buzzer of 0.5 Hz.
c. The system is activated and dormant with signals of two blinks (blink movement within 2 s).
d. Various data base of different participant should be collected for data training progress.

Supplementary Note 3:

Basic setup of the eye-controlled input modality.

a. The deep learning progress extract data of the electrostatic signal every 2 s, and every eye movement of the participant are required to finished within 2 s, which is reminded by a buzzer of 0.5 Hz.

b. Signals of single blink is ignored because in the most time, single blink is an unconscious movement of human.

c. For overcome interference of unconscious eyeball rotation, signals of whole wide-angle rotation (a round rotation, for example, eyeball rotates to up and backs to the center) are induced to controlled the moving direction of the mouse.

d. The moving distance of the mouse cursor is set in constant, up and down moving distance is the same quantity, as well as the left and right distance. In addition, the distance of one direction can also be set according to various degrees of eyeball rotations but deep learning with training data is necessary.

e. Various data base of different participant should be collected for data training progress.

Reference

1. López, A., Fernández, M., Rodríguez, H., Ferrero, F., Postolache, O. Development of an eog-based system to control a serious game. *Measurement* **127**, 481-488 (2018).
2. Rostaminia, S., Lamson, A., Maji, S., Rahman, T., Ganesan, D. W!Nce: Eyewear solution for upper face action units monitoring. *Etra 2019: 2019 Acm Symposium on Eye Tracking Research & Applications*, (2019).
3. Homayounfar, S. Z. et al. Multimodal smart eyewear for longitudinal eye movement tracking. *Matter* **3**, 1275-1293 (2020).
4. Lee, J. H. et al. 3d printed, customizable, and multifunctional smart electronic eyeglasses for wearable healthcare systems and human-machine interfaces. *ACS Appl. Mater. Interfaces* **12**, 21424-21432 (2020).
5. Diaz-Romero, D. J., Rincon, A. M. R., Miguel-Cruz, A., Yee, N., Stroulia, E. Recognizing emotional states with wearables while playing a serious game. *IEEE Trans. Instrum. Meas.* **70**, 1-12 (2021).
6. Xu, J. et al. Electrooculography and tactile perception collaborative interface for 3d human-machine interaction. *ACS Nano* **15**, (2022).
7. Kireev, D. et al. Multipurpose and reusable ultrathin electronic tattoos based on $PtSe_2$ and $PtTe_2$. *ACS Nano* **15**, 2800-2811 (2021).
8. Kireev, D. et al. Fabrication, characterization and applications of graphene electronic tattoos. *Nat. Protoc.* **16**, 2395-2417 (2021).
9. Ameri, S. K. et al. Imperceptible electrooculography graphene sensor system for human–robot interface. *npj 2D Mater. Appl.* **2**, (2018).
10. Wan, S. et al. Highly stretchable starch hydrogel wearable patch for electrooculographic signal detection and human–machine interaction. *Small Struct.* **2**, (2021).
11. Sakthivelpathi, V. et al. Capacitive eye tracker made of fractured carbon nanotube-paper composites for wearable applications. *Sensors Actuat. A-Phys.* **344**, (2022).
12. Lee, S. et al. Ultrathin nanogenerators as self-powered/active skin sensors for tracking eye ball motion. *Adv. Funct. Mater.* **24**, 1163-1168 (2014).
13. Pu, X. et al. Eye motion triggered self-powered mechnosensational communication system using triboelectric nanogenerator. *Sci. Adv.* **3**, e1700694 (2017).
14. Vera Anaya, D., He, T., Lee, C., Yuce, M. R. Self-powered eye motion sensor based on triboelectric interaction and near-field electrostatic induction for wearable assistive technologies. *Nano Energy* **72**, (2020).
15. Kim, M. P., Kim, Y.-R., Ko, H. Anisotropic silver nanowire dielectric composites for self-healable triboelectric sensors with multi-directional tactile sensitivity. *Nano Energy* **92**, (2022).
16. Zhu, J. et al. Triboelectric patch based on maxwell displacement current for human energy harvesting and eye movement monitoring. *ACS Nano* **16**, (2022).
