# Supplementary material for: Eye tracking and eye expression decoding based on transparent, flexible and ultra-persistent electrostatic interface
Source: Nat Commun. 2023 Jun 7;14:3315. doi: 10.1038/s41467-023-39068-2 (PMC10247702; doi:10.1038/s41467-023-39068-2)
Supplement: Supplementary file 3 — Description to Additional Supplementary Information [file 41467_2023_39068_MOESM3_ESM.pdf]

Description of Additional Supplementary Files

Supplementary Movie 1. Eye movement patterns.

Supplementary Movie 2. Closing eye tracking.

Supplementary Movie 3. Eye tracking system for visual preference analysis.

Supplementary Movie 4. Eyecontrolled input modality
